# Nutrition and Sarcopenia: A Review of the Evidence and Implications for Preventive Strategies

**DOI:** 10.1155/2012/510801

**Published:** 2012-03-15

**Authors:** Siân Robinson, Cyrus Cooper, Avan Aihie Sayer

**Affiliations:** MRC Lifecourse Epidemiology Unit (University of Southampton), Southampton General Hospital, Southampton SO16 6YD, UK

## Abstract

Prevention of age-related losses in muscle mass and strength is key to protecting physical capability in older age and enabling independent living. To develop preventive strategies, a better understanding is needed of the lifestyle factors that influence sarcopenia and the mechanisms involved. Existing evidence indicates the potential importance of diets of adequate quality, to ensure sufficient intakes of protein, vitamin D, and antioxidant nutrients. Although much of this evidence is observational, the prevalence of low nutrient intakes and poor status among older adults make this a current concern. However, as muscle mass and strength in later life are a reflection of both the rate of muscle loss and the peak attained in early life, efforts to prevent sarcopenia also need to consider diet across the lifecourse and the potential effectiveness of early interventions. Optimising diet and nutrition throughout life may be key to preventing sarcopenia and promoting physical capability in older age.

## 1. Introduction

Sarcopenia is the loss of muscle mass and strength that occurs with advancing age [1]. Although definitions (and therefore estimates of prevalence) vary, it is widely recognised as a common condition among older adults, and one that is associated with huge personal and financial costs [1, 2]. Declining muscle mass and strength are expected components of ageing. However, the rate of decline differs across the population [1, 3], suggesting that modifiable behavioural factors such as diet and lifestyle may be important influences on muscle function in older age. This paper considers the evidence that links diet to muscle mass and strength, and implications for strategies to prevent or delay sarcopenia in older age.

## 2. Nutrition and Ageing

Food intake falls by around 25% between 40 and 70 years of age [4]. In comparison with younger ages, older adults eat more slowly, they are less hungry and thirsty, consume smaller meals, and they snack less [4]. The mechanisms for the “anorexia of ageing” are not fully understood but there may be a range of physiological, psychological, and social factors that influence appetite and food consumption, including loss of taste and olfaction, increased sensitivity to the satiating effects of meals, chewing difficulties, and impaired gut function [4, 5]. The negative consequences of these changes are compounded by the effects of functional impairments that impact on the ability to access and prepare food, psychological problems such as depression and dementia, as well as the social effects of living and eating alone. Low food intakes and monotonous diets put older people at risk of having inadequate nutrient intakes [6]. Thus in a vicious cycle, declining muscle strength and physical capability in older age may increase the risk of poor nutrition, whilst poor nutrition may contribute to further declines in physical capability.

The exact estimates of the prevalence of poor nutrition may differ according to the definitions used, but studies of community-dwelling adults consistently suggest that it is common in older age. For example in the National Diet and Nutrition Survey in the UK, 14% of older men and women living in the community, and 21% of those living in institutions, were at medium or high risk of undernutrition [7]. Estimates of the prevalence of undernutrition in older patients admitted to hospital are even greater, ranging up to 72% [8, 9]. These figures are clearly substantial and indicate that there are significant numbers of older adults living in developed settings who currently have less than optimal nutrition.

## 3. Is Diet a Modifiable Influence on Sarcopenia?

There are two consequences of declining food intakes in older age that could be important for muscle mass and strength. Firstly, lower energy intakes, if not matched by lower levels of energy expenditure, lead to weight loss, including a loss of muscle mass [4]. Secondly, as older people consume smaller amounts of food, it may become more challenging for them to meet their nutrient needs—particularly for micronutrients. For older people with low food intakes, this highlights the importance of having diets of adequate quality. Although the importance of adequate nutrition has been recognised for a long time, its contribution to muscle mass and strength has not been studied extensively and much of the research in this area is relatively new [10]. A number of interventions have been studied, ranging from provision of nutritional support [11], to supplementation with specific nutrients [12, 13]. The nutrients that have been most consistently linked to sarcopenia and frailty in older adults are vitamin D, protein, and a number of antioxidant nutrients, that include carotenoids, selenium, and vitamins E and C [10]. However, there is also some evidence that variations in long-chain polyunsaturated fatty acid status may have important effects on muscle strength in older people [13].

### 3.1. Protein

Protein is considered a key nutrient in older age [14]. Dietary protein provides amino acids that are needed for the synthesis of muscle protein, and importantly, absorbed amino acids have a stimulatory effect on muscle protein synthesis after feeding [15]. There is some evidence that the synthetic response to amino acid intake may be blunted in older people, particularly at low intakes [14], and when protein is consumed together with carbohydrate [16]. Recommended protein intakes may, therefore, need to be raised in older people in order to maintain nitrogen balance and to protect them from sarcopenic muscle loss [14].

Whilst there is currently no consensus on the degree to which dietary protein requirements change in older age, there is important observational evidence that an insufficient protein intake may be an important contributor to impaired physical function. For example, in the US Health, Aging and Body Composition Study, a greater loss of lean mass over 3 years, assessed using dual-energy X-ray absorptiometry, was found among older community-dwelling men and women who had low energy-adjusted protein intakes at baseline [17]. The differences were substantial, such that the participants with protein intakes in the top fifth of the distribution lost 40% less lean mass over the follow-up period when compared with those in bottom fifth. Protein and/or amino acid supplementation should, therefore, have the potential to slow sarcopenic muscle loss. However, whilst amino acid supplementation has been shown to increase lean mass and improve physical function [18], other trials have not been successful [16, 19]. Further work, including longer-term trials, is needed to define optimal protein intakes in older age [16].

### 3.2. Vitamin D

An association between vitamin-D-deficient osteomalacia and myopathy has been recognised for many years [20], but the role of vitamin D, and the extent to which it has direct effects on normal muscle strength and physical function remains controversial [21]. The potential mechanisms that link vitamin D status to muscle function are complex and include both genomic and nongenomic roles [20, 22]. The vitamin D receptor (VDR) has been isolated from skeletal muscle, indicating that it is a target organ [20], and polymorphisms of the VDR have been shown to be related to differences in muscle strength [23]. At the genomic level, binding of the biologically active form of the vitamin (1,25-dihydroxyvitamin D) results in enhanced transcription of a range of proteins, including those involved in calcium metabolism [20]. The nongenomic actions of vitamin D are currently less well understood [22].

Much of the epidemiological literature is consistent with the possibility that there are direct effects of vitamin D on muscle strength. For example, among men and women aged 60 years and older in NHANES III, low vitamin D status (serum 25-hydroxyvitamin D < 15 ng mL^−1^) was associated with a fourfold increase in risk of frailty [24], and in a meta-analysis of supplementation studies of older adults, Bischoff-Ferrari et al. [12] showed that supplemental vitamin D (700–1000 IU per day) reduced the risk of falling by 19%. However, the evidence is not always consistent as some observational studies find no association between vitamin D status and physical function, and supplementation studies have not always resulted in measurable improvements in function [21]. In a review of published studies, Annweiler and colleagues [21] discuss the reasons for the divergence in study findings, some of which may be due to methodological differences, including a lack of consideration of confounding influences in some studies. Further evidence is needed, particularly as vitamin D insufficiency is common among older adults [24].

### 3.3. Antioxidant Nutrients

There is increasing interest in the role of oxidative stress in aetiology of sarcopenia, and markers of oxidative damage have been shown to predict impairments in physical function in older adults [25]. Damage to biomolecules such as DNA, lipid, and proteins may occur when reactive oxygen species (ROS) are present in cells in excess. The actions of ROS are normally counterbalanced by antioxidant defence mechanisms that include the enzymes superoxide dismutase and glutathione peroxidase, as well exogenous antioxidants derived from the diet, such as selenium, carotenoids, tocopherols, flavonoids, and other plant polyphenols [15, 25]. In older age, an accumulation of ROS may lead to oxidative damage and contribute to losses of muscle mass and strength [15].

A number of observational studies have shown positive associations between higher antioxidant status and measures of physical function [10]. Importantly these associations are seen both in cross-sectional analyses and in longitudinal studies, such that poor status is predictive of decline in function. The observed effects are striking. For example, among older men and women in the InCHIANTI study, higher plasma carotenoid concentrations were associated with a lower risk of developing a severe walking disability over a follow-up period of 6 years; after taking account of confounders that included level of physical activity and other morbidity, the odds ratio was 0.44 (95% CI 0.27–0.74) [26]. Inverse associations have also been described for vitamin E and selenium status and risk of impaired physical function [10]. There have been few studies of older adults to determine how antioxidant supplementation affects muscle strength, and the benefits of supplementation remain uncertain [27]. Since ROS have both physiological and pathological roles, interventions based on simple suppression of their activities may be unlikely to improve age-related declines in muscle mass and function [28]. However, low antioxidant intakes and status are common [6, 29], and this remains an important question to be addressed.

### 3.4. Long-Chain Polyunsaturated Fatty Acids (LCPUFAs)

Sarcopenia is increasingly recognised as an inflammatory state driven by cytokines and oxidative stress [30]. Since eicosanoids derived from 20-carbon polyunsaturated fatty acids are among the mediators and regulators of inflammation [13], this raises the possibility that variations in intake of n-3 and n-6 LCPUFAs, and their balance in the diet, could be of importance. In particular, n-3 LPUFAs have the potential to be potent anti-inflammatory agents [13]. There is some observational evidence to support an effect of n-3 LCPUFA status on muscle function, as higher grip strength was found in older men and women who had greater consumption of oily fish [31]—one of the richest sources of n-3 LCPUFAs in the UK diet. Consistent with this finding, a number of studies of patients with rheumatoid arthritis have shown that supplementation with fish oil resulted in improved grip strength [13]. In a recent randomised controlled trial, supplementation of older adults with n-3 LCPUFA (eicosapentaenoic and docosahexaenoic acids) resulted in an enhanced anabolic response to amino acid and insulin infusion. Whilst these novel data suggest that the stimulation of muscle protein synthesis by n-3 LCPUFA supplementation could be useful for the prevention and treatment of sarcopenia [32], further evidence is needed to establish the therapeutic potential of n-3 LCPUFAs in inflammatory conditions [13].

### 3.5. Foods and Dietary Patterns

One problem with the existing evidence base is that dietary components are often highly correlated with each other. This may help to explain why the effects of supplementation with single nutrients may be less than that predicted by the observational evidence. It also means that from observational data it may be difficult to understand the relative importance of the influences of different nutrients on sarcopenia. For example, whilst an antioxidant nutrient such as *β*-carotene may be causally related to variations in physical function, it may also be acting as a marker of other components of fruit and vegetables. In turn, since diets are patterned, high fruit and vegetable consumption may be indicators of other dietary differences which could be important for muscle function, such as greater consumption of oily fish and higher intakes of vitamin D and n-3 LCPUFAs [33]. The cumulative effects of nutrient deficiencies have been described by Semba et al. [34], in which he estimated that each additional nutrient deficiency raised the risk of frailty in older women by almost 10%. This emphasises the importance of the quality of diets of older adults, as well as the quantity of food consumed, to ensure that intakes of a range of nutrients are sufficient.

Compared with the evidence that links variations in nutrient intake and status to physical function, much less is known about the influence of dietary patterns and dietary quality in older age. “Healthy” diets, characterised by greater fruit and vegetable consumption, wholemeal cereals, and oily fish, have been shown to be associated with greater muscle strength in older adults [31]. Data from studies of younger adults appear to be consistent with this finding. For example, among women aged 42–52 years, “unhealthy” diets, characterised by higher saturated fat intakes and lower fruit and vegetable consumption, were associated with greater functional limitations over a 4-year follow-up period [35]. Benefits of healthier diets and greater fruit and vegetable consumption on physical function in mid-life have also been described in women in the Whitehall study [36], and in men and women in the Atherosclerosis Risk in Communities Study [37]. Intervention studies that take a food-based or “whole diet” approach are likely to change intakes of a range of nutrients and, therefore, have the potential to be more effective than single nutrient supplementation studies in preventing age-related losses in muscle mass and strength.

### 3.6. Diet and Exercise

Resistance exercise training interventions have been shown to be effective in increasing muscle strength and improving physical function in older adults [38]. A further issue in understanding a possible protective role for diet in sarcopenia is, therefore, the potential for interactions between diet and exercise, and the extent to which interventions that combine supplementation and exercise training may be more effective than changing nutrient intake alone. The interactive effects of diet and exercise on physical function have been studied most extensively in relation to protein/amino acid supplementation. For example, whilst consumption of a high protein meal has been shown to increase muscle protein synthesis in older adults by ~50%, combining a high protein meal with resistance exercise increases synthesis more than 100% [39]. However, a number of studies of older adults have failed to show additional benefits of protein/amino acid supplementation on the skeletal muscle response to prolonged resistance exercise training [15, 40], and the implications for long-term effects of combined exercise training and high protein intakes are, therefore, not clear [16]. Current findings point to the need for further research—particularly to address the effects of differing quantity and timing of supplementation [39, 40]. At present we have limited insights into the combined effects of vitamin D supplementation and resistance exercise on muscle strength and function [41]. 

## 4. Lifelong Nutrition and Sarcopenia

An important limitation to the current evidence base that links nutrition to sarcopenia is that much of the observational data are from cross-sectional studies. Aside from methodological considerations of studying older adults who may have a number of comorbidities, this raises particular issues that may limit our understanding of the potential importance of the role of nutrition in the loss of muscle mass and strength with age.

Firstly, the health of older people is influenced by events throughout their lives [10], and achievement of optimal function may, therefore, depend on lifelong exposure to a healthy diet and lifestyle. Although there is evidence that healthier eating behaviours are reasonably stable in adult life [42], little is known of changes in dietary habits in older people, at a time when morbidity-related dietary advice is available, and lifestyle may be changing rapidly. The influence of lifelong nutrition on age-related changes in muscle mass and strength has been little studied, but in terms of interventions to delay or prevent sarcopenia in older age, there may be key opportunities earlier in the lifecourse that need to be recognised.

**Figure 1:**
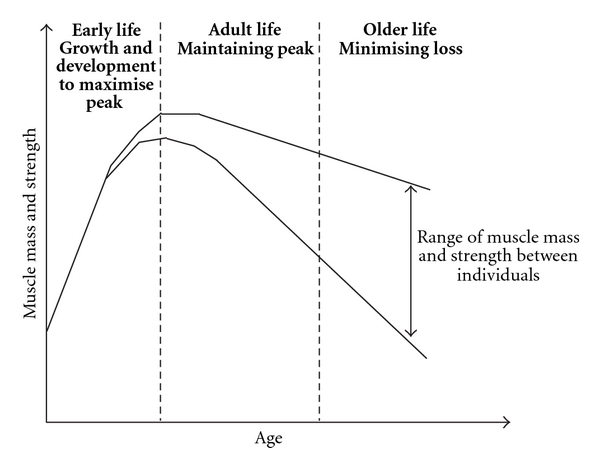
A lifecourse model of sarcopenia (from [43]).

A second consideration is that muscle mass and strength achieved in later life are not only determined by the rate of muscle loss, but also reflect the peak attained in early life (Figure 1, [43]). Thus, factors that influence growth, such as variations in early nutrition, may contribute to muscle mass and strength in older age.

A key finding, that highlights the importance of lifecourse influences, is that low weight at birth predicts lower muscle mass and strength in adult life. This is a consistent finding across a number of studies [44]. Although little is currently known about the influence of diet in early life on sarcopenia, recent studies of adolescents have provided evidence of nutrient effects on muscle mass and function earlier in the lifecourse. Consistent with studies of older adults, low vitamin D status has been shown to be associated both with lower grip strength and with poorer muscle power and velocity [45, 46]. However, randomized controlled trials of vitamin D supplementation of adolescents have had mixed results. Among premenarcheal girls who were supplemented with vitamin D over 1 year, there were graded increases in lean mass, although supplementation did not result in measurable differences in grip strength [47]. In contrast, vitamin D supplementation of adolescent boys and postmenarcheal girls has not been shown to be effective in increasing lean mass or muscle strength or power [47, 48]. Ward et al. [48] conclude that earlier interventions, before the period of peak muscle mass accretion, may be needed to improve muscle function and physical performance.

To date, few studies have examined the role of diet in early childhood in the acquisition of muscle mass and effects on later function, although there is some evidence that it could be important. For example, the risk of frailty has been shown to be greater in older adults who grew up in impoverished circumstances, and who experienced hunger in childhood [49]. However, animal models suggest that nutrition even earlier in life may be key, as muscle growth in the neonatal period is highly sensitive to variations in nutrient intake [50]. In two recent studies, the role of variations in infant diet has been addressed, but with differing results. Among children in the ALSPAC study, duration of breastfeeding was not associated with physical work capacity assessed at the age 9 years [51], whilst in adolescents studied in the HELENA cohort, longer duration of breastfeeding was associated with measurable differences in physical performance–particularly in lower body explosive strength [52]. Consistent with these latter findings, longer duration of breastfeeding and greater compliance with infant feeding guidance has been shown to be associated with greater lean mass in later childhood [53]. Dietary patterns “track” across childhood [54], and this may simply reflect continuing benefits of healthier diets. However, it does suggest that variations in early postnatal diet could have implications for muscle function in later life.

We currently know little about the contribution of nutrition across the lifecourse to muscle mass and strength in adult life, and further work is needed to understand how early nutrition influences the acquisition of peak muscle mass, and the role played by nutrition in the trajectory of age-related losses in muscle function. Taking a lifecourse approach to understanding the links between nutrition and muscle mass and function in older age could change dietary strategies to prevent sarcopenia in the future.

## 5. Conclusion

To develop strategies to prevent or delay sarcopenia, a better understanding is needed of the lifestyle factors that influence the rate of decline of muscle mass and strength in older age, and the mechanisms involved. Existing evidence indicates the potential importance of diets of adequate quantity and quality, to ensure sufficient intakes of protein, vitamin D, and antioxidant nutrients. Although much of this evidence is observational and the mechanisms are not fully understood, the high prevalence of low nutrient intakes and poor status among older adults make this a current concern. However, muscle mass and strength achieved in later life are not only determined by the rate of muscle loss, but also reflect the peak attained earlier in life, and efforts to prevent sarcopenia also need to recognise the potential effectiveness of interventions earlier in the lifecourse. Optimising diet and nutrition throughout life may be key to preventing sarcopenia and promoting physical capability in older age.
